# Mass Hysteria among Beneficiary Students of the School-Feeding Program in Addis Ababa, Ethiopia

**DOI:** 10.4314/ejhs.v32i3.12

**Published:** 2022-05

**Authors:** Solomie Jebessa, Handsome Deksiso, Muluwork Tefera, Yonas Bahretibeb

**Affiliations:** 1 Pediatrics Infectious Disease Specialist St. Paul's Hospital Millennium Medical College, Addis Ababa, Ethiopia; 2 Department of Pediatrics and Child Health, Tikur Anbessa Specialized Hospital. Addis Ababa, Ethiopia; 3 Department of Pediatrics and Child Health, Tikur Anbessa Specialized Hospital Addis Ababa, Ethiopia; 4 Department of Psychiatry, Tikur Anbessa Specialized Hospital. Addis Ababa, Ethiopia

**Keywords:** Mass hysteria, factors attributable, School feeding, Conversion disorder, Somatic symptom disorder

## Abstract

**Background:**

Mass hysteria is described as the rapid spread of conversion disorder without organic basis among a group of people. Mass hysteria can occur in work place and commonly in schools. There are usually some factors attributable to the episode; however, the lack of a pathogen upon investigation is a fundamental characteristic. We are reporting an episode of mass hysteria from two schools in Addis Ababa, Ethiopia.

**Methods:**

Clinical record, laboratory investigation, toxicology study from the food and psychiatric evaluations.

**Results:**

On November 25, 2019 a total of 113 students were brought from two schools in Addis to Tikur Anbessa Specialized Hospital. Most were between the ages of 10 and 15 years and were female students. Their school breakfast of bread and marmalade was attributed as the cause of the episode; however only 49% of the students brought in had eaten the food. The majority complained of nausea and vomiting but most had normal physical finding; and their symptoms were resolved without treatment. The laboratory investigation on samples of blood and stool were negative for bacterial growth and food culture and toxicology were non-revealing. Most were reassured and few were given symptomatic treatment.

**Conclusion:**

This mass hysteric episode is similar to episodic reports from other school feeding programs in some Asian and African countries. The finding of this report is important for health care practitioners to consider mass psychogenic illness in case they face similar mass presentation without objective finding; and will help to avoid unnecessary costly investigations.

## Introduction

Hysteria is defined as a constellation of symptoms suggestive of physical illness with no identifiable cause and little evidence of disease (1). Mass hysteria is described as the appearance of these symptoms among a group of people (2).

In the Diagnostic and Statistical Manual of Mental Disorders (DSM-5), symptoms that were once labeled under the broad umbrella of Hysteria would fit under what is now referred to as somatic symptom disorder. Somatic symptom disorder involves having a significant focus on physical symptoms such as weakness, pain or shortness of breath. This preoccupation with symptoms is so pronounced that it results in significant distress and difficulties with normal functioning. It is important to note that this does not involve faking an illness; whether the person is sick or not, they *believe* that they are ill (3). In this paper we prefer to use the terminology of “Mass Hysteria” as most of the previous episodes are recorded with similar name.

The commonalities in most Mass Hysteria events are fear, sadness, anxiety, problematic family issues, and death of someone close (4, 5). The behaviors or symptoms recorded during Mass Hysteria outbreaks include convulsion, abnormal body movement, trembling, amnesia, abdominal pains, chest tightness, dizziness, fainting, crying loudly, falling, rolling, headaches, hyperventilation, nausea, vomiting, refusal to eat, withdrawal, hallucinating, hypersensitivity to noise, palpitation, anxiety, hiccups, laughing, tics, attacking others and screaming. (5,6,7,8). In one South African school, generalized itching upon entering the school was observed (9)

Mass Hysteria is common among the preadolescent to adolescent age group and in females. It has been observed in schools, factories and work places; and 60% of literatures recorded occurred in schools (6, 7, 10, 11–14, 15). Mass Hysteria has been also common among developing nations: some of the episodes were associated with events like mass deworming observed at schools in Zamboanga peninsula, Philippines (13), following a school feeding program in Northwest Bangladesh (14), as well as similar episodes observed in Nepal (15). Various Mass Hysteria episodes have been observed in African schools: from South Africa, Malawi, Zimbabwe, Zambia and Democratic Republic of Congo (4, 16,17).

In Ethiopia, the first episode of mass hysteria was in Gondar city in 1982, and other mass hysteria episodes occurred in Bati high school (18) and in Kombolcha, a town in northwest Ethiopia. Separation from the school environment for 1–2 weeks resolved the episode (19).

The impact of mass hysteria in schools is very significant, causing postponement of exams, psychological impact on the affected students and parents, as well as temporary school closure. The lack of a pathogen upon investigation of the illness is a primary characteristic of all mass hysteria episodes. The investigations may include the environment, physical finding on the cases, investigations of food samples, patient body fluid, and urine and stool samples, depending on their importance (6).

The psychosocial environment, beliefs, and spiritual events play important role in the spread of mass hysteria. Symptoms often resolve after the subjects are separated from each other or removed from the environment and after reassurance that the illness have been resolved (7,20).

The manner with which an outbreak is handled is important to consider: the respective public health department has to be involved in deciding how and what to disclose to the general population. This is because speculated or twisted information will be more damaging than the event per se, and sometimes may also trigger symptoms in the nearby similar group of population (21). On the other hand, in some mass hysteria events, parents and the media do not accept the diagnosis, which further adds stress on the children to return back to school (4).

A school feeding program started in Addis Ababa, Ethiopia in September 2019 with strong commitment and political will from the government. The incident we are reporting occurred in two of the schools in Addis Ababa on November 25, 2019. We believe that reporting this episode will create better awareness among health care providers, school communities, parents, and to the population at large.

## Methods

Descriptive report of the incident including the medical history and physical findings, laboratory investigation such as: blood culture, stool culture, food culture, food toxicology were conducted. Additionally psychiatric evaluation was done for few students by psychiatrist when referred by the pediatrician.

## Results

**Figure 1:**
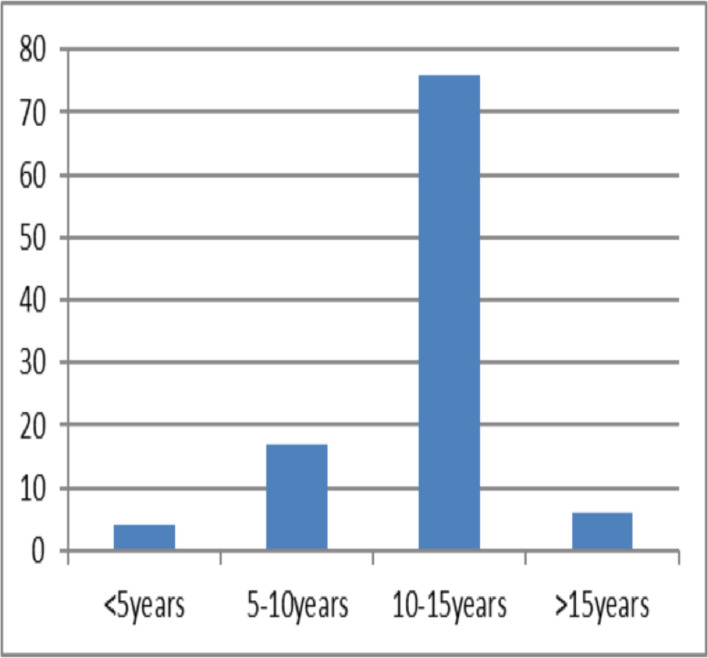
Age distribution of students who came with mass hysteric episodes (n=103) seen at Tikur Anbessa Specialized Hospital, Addis Ababa, Nov 25, 2019.

On November 25, 2019, a total of 113 students from two schools were brought by the school principals and teachers to Tikur Anbessa Specialized Hospital at Addis Ababa. Patient record charts were issued for 103 of them. All of the students were seen at the pediatrics emergency unit and three were referred to psychiatry department for better evaluation. Senior pediatricians, pediatrics residents, medical interns and nurses participated in the medical evaluation and treatment of the students. Their age range was between 3.5 to 17 years with the majority (73%) being between 10–15 years (Figure 1). Sixty-five students (62.1%) were girls.

The majority (69.9%) of the students came because they were sick, while the rest (30.1%) came because the school sent them for medical evaluation. Their breakfast meal consisting of bread with marmalade was attributed as the cause of the symptoms. Nearly half (49%) of students reported that they ate the breakfast at school that day, while 15% did not eat, and for 36% it was not recorded whether they ate or not.

**Figure 2:**
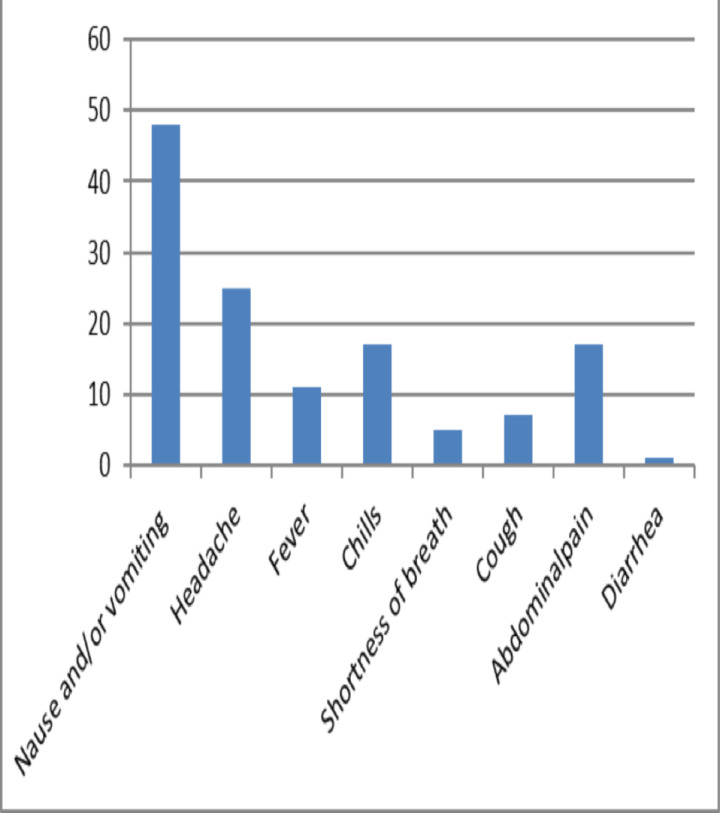
Presenting compliant of students who came with mass hysteric episodes (n=103) seen at Tikur Anbessa Specialized Hospital, Addis Ababa, Nov 25, 2019.

The majority (46%) of the students presented with complaints of nausea and vomiting, 24.4% complained of a headache, 16% complained of abdominal pain, 16% complained of chills, 10% complained of having a fever, 6.8% had cough, and 4.9% complained that they had shortness of breath (Figure 2).

Binary logistic regression analysis of children who ate the breakfast with the complaints of nausea and vomiting was not statistically significant (P value of 0.085).

Upon physical examination, 18.4% had tachycardia, 6.8% had tachyponea, 4.9% had recordable fever, and one patient had carpal spasm which was secondary to low calcium level in the blood (hypocalcaemia) following hyperventilation.

On interim assessment, 60% of the students (61) were assessed as normal (stable), 30% were diagnosed with probable food poisoning, 3% with acute gastroenteritis, 1% with hypovolemic shock, 3% with hyperventilation and anxiety, 2% with pneumonia and 1% with acute febrile illnesses. Blood and stool samples were sent for laboratory investigations. Blood culture was done for 5 of the symptomatic students, whereas stool culture was done for 4 of them. All results showed no growth on cultures. Laboratory investigations done at EPHI of the food samples (marmalade and bread) were also negative for pathogens (on cultures) or toxins.

Of those who were diagnosed (N = 42) with disease entity majority 71.8 % were reassured, 14.6% received Intravenous (IV) normal saline, while 7.8% received both IV normal saline and ceftriaxone. 2% received ORS, 1% received IV ceftriaxone, 2% received oral ciprofloxacin, and one patient was treated with IV calcium gluconate.

## Discussion

School feeding programs were started in Addis Ababa in September 2019 to support the nutrition of students from low-income families and thus to improve their performance and decrease dropping out rates from school. It is imperative that such kinds of mass feeding programs may be challenged with tangible and non-tangible problems. This descriptive study reports the clinical evaluation findings of students from two of the schools in Addis Ababa who were brought to Tikur Anbessa Specialized Hospital on November 25, 2019. The symptoms rapidly spread following the symptoms of the first student who had abdominal pain, nausea, vomiting and fainting after consuming the morning meal of bread with marmalade.

Similar outbreaks have been reported among beneficiaries of school feeding programs in Northwest Bangladesh in 2010; in which an outbreak of abdominal pain, heart burn and bitter test was reported by the students who ate high energy biscuits supplied by the school. They identified no organism on stool samples and none the biscuits supplied were rancid (14). Acute illness in school with no objective finding and no identifiable environmental risk should lead to the diagnosis of Mass Hysteria.

In our study, majorities (62.1%) of the students were female and most were adolescents between the age of 10–15 years. A majority had GI symptoms which were also seen in other African reports. In August 2002, at a primary school in Central South Africa, 27 children lost consciousness suddenly; most were females, the other manifestations were similar to our report including stomach cramps, nausea and chest tightness (6). In another report from a school in Tanzania in 2008, 20 students had slumming over their exam paper and collapsing (6).

Similarly, our findings correlated with the findings of case control study by Worku Lake, Mastewal, in Kombolcha primary school. All affected were females and adolescents (9–16 years) and had presenting symptoms of headache, shouting, crying, followed by fainting, nausea, epigastric pain and cramp (19).

Our patients' parameters were also similar with the study finding by Beyene et.al in April 10, 2010 in Bati high school, in which all affected were female with median age of 16 years. In contrast to our report those groups presented with shortness of breath, fear and crying, anxiety and inability to move limbs (18).

The limitation of our study was that we did not have documented socio-demographic profiles (living conditions, family income etc.) of the patients in this study; future studies should evaluate the socioeconomic background of patients presenting with similar complaint.

Mass hysteria is common phenomenon in school and school feeding programs. The finding of this report is important for health care practitioners to consider Mass Hysteria in case they face mass presentation without objective finding. Considering this diagnosis, it is important to design management and avoid unnecessary and costly investigations.
